# Finite element model of ocular adduction with unconstrained globe translation

**DOI:** 10.1007/s10237-023-01794-3

**Published:** 2024-02-28

**Authors:** Somaye Jafari, Joseph Park, Yongtao Lu, Joseph L. Demer

**Affiliations:** 1 Stein Eye Institute, UCLA, University of California , 100 Stein Plaza, Los Angeles, CA 90095-7002 USA; 2 Bioengineering Department, University of California, Los Angeles, USA; 3 Department of Engineering Mechanics, Dalian University of Technology, Dalian, China; 4 Neuroscience Interdepartmental Program, University of California, Los Angeles, USA; 5 Department of Neurology, University of California, Los Angeles, USA

**Keywords:** Eye, Finite element model, Muscle contraction, Eye movement, Suspensory system

## Abstract

Details of the anatomy and behavior of the structures responsible for human eye movements have been extensively elaborated since the first modern biomechanical models were introduced. Based on these findings, a finite element model of human ocular adduction is developed based on connective anatomy and measured optic nerve (ON) properties, as well as active contractility of bilaminar extraocular muscles (EOMs), but incorporating the novel feature that globe translation is not otherwise constrained so that realistic kinematics can be simulated. Anatomy of the hemisymmetric model is defined by magnetic resonance imaging. The globe is modeled as suspended by anatomically realistic connective tissues, orbital fat, and contiguous ON. The model incorporates a material subroutine that implements active EOM contraction based on fiber twitch characteristics. Starting from the initial condition of 26° adduction, the medial rectus (MR) muscle was commanded to contract as the lateral rectus (LR) relaxed. We alternatively modeled absence or presence of orbital fat. During pursuit-like adduction from 26 to 32°, the globe translated 0.52 mm posteriorly and 0.1 mm medially with orbital fat present, but 1.2 mm posteriorly and 0.1 mm medially without fat. Maximum principal strains in the optic disk and peripapillary reached 0.05–0.06, and von-Mises stress 96 kPa. Tension in the MR orbital layer was ~ 24 g-force after 6° adduction, but only ~ 3 gm-f in the whole LR. This physiologically plausible simulation of EOM activation in an anatomically realistic globe suspensory system demonstrates that orbital connective tissues and fat are integral to the biomechanics of adduction, including loading by the ON.

## Introduction

### Mechanical loading on the eye

Ocular movements have historically been simplified as pure rotations caused by torques generated by extraocular muscle (EOM) tensions applied exclusively to the eyeball, and balanced by elastic recoil of passive connective tissues while neglecting the optic nerve (ON) altogether. It was more recently recognized that the EOMs distribute a substantial fraction of their forces to moving a gimbal system of connective tissues upon which they insert (Demer 1995, 2004, 2006a, 2006b, 2007) and that the ON can act as a significant mechanical load on the eye (Demer 2016). These fundamental anatomical and functional discoveries now necessitate elaboration of existing quantitative models of ocular mechanics to make them relevant for understanding eye movements in health and disease.

### Models of the ocular motor apparatus

Orbital geometry, connective tissues, EOMs, and their innervations interact with complexity that necessitates implementation of quantitative models for insight (Miller and Robinson 1984). Robinson devised an early lumped parameter model based on balance of static forces of EOM’s and orbital tissues (Robinson 1975). This model was elaborated by Miller and Robinson to include EOM paths, globe translation, and binocularity (Miller and Robinson 1984), allowing simulation of some forms of strabismus and strabismus surgery. Related lumped models implementing EOMs as single compartments have been proposed (France and Burbank 1979; Kault et al. 1989; Simonsz and den Tonkelaar 1990). These lumped parameter models have provided many insights, and motivated much quantitative research.

However, the understanding of the structure and function of the ocular motor apparatus has evolved significantly since the emergence of these early models. Functional imaging and histological studies have demonstrated existence of two forms of compartmentalization in EOMs: the first, embodied in the active pulley hypothesis, provides that the orbital layer (OL) of each EOM inserts on and translates the EOM’s connective tissue pulley, while the global layer (GL) exerts oculorotary torque (Demer 2004; Kono et al. 2002). The second form, embodied in the transverse compartmentalization concept that differential tension can occur in the two halves of broad EOM tendon insertions (Demer 2015; Shin et al. 2014), permits each EOM to exert torque in directions transverse to their lengths to participate in vergence (Demer and Clark 2014) and the vestibulo-ocular reflex (Clark and Demer 2012a). A recent lumped parameter model incorporates these features using separate string primitives for the OL and both GL compartments, with pulleys implemented as elastically suspended tubes encircling the EOMs (Wei et al. 2022). While this string model permits exploration of possible EOM innervation and tension sets, it does not attempt to evaluate stress and strain in actual anatomical regions, nor does it incorporate the ON. Another recent physiological advance is recognition that the globe translates systematically as it rotates, effectively rolling from side to side while rotating horizontally (Demer and Clark 2019). Globe translation has rarely been incorporated in lumped parameter models.

### Finite element models (FEMs)

Finite element models (FEMs) of orbital biomechanics emerged after most lumped parameter models and can provide greater anatomical realism than lumped parameter models. The early FEM of Schutte et al. implemented small eye rotations using a thermal expansion algorithm to change EOM lengths, while constraining EOM paths by their intrinsic stiffness and relatively homogeneous, non-muscular orbital contents that these authors broadly considered to be “orbital fat” (Schutte et al. 2006). Schutte et al. assumed the sclera and Tenon’s capsule to be tied to orbital fat (Schutte et al. 2006), while in the real orbit, the sclera slides over the fat. This pioneering FEM was limited both by its anatomical simplifications, including unicompartmental EOMs and absence of pulleys, as well as by maximal strains arising in its elements. Wang et al. used FEM to predict strain in the ON head during horizontal eye rotation; however, the globe center was fixed, and arbitrary rotational force was applied to the globe rather than by physiological EOM contraction (Wang et al. 2016). Shin et al. developed a FEM of the human orbit incorporating bovine tissue properties to evaluate tethering traction exerted by the ON in large adduction (Shin et al. 2017). This model also assumed ocular rotation about a fixed center, again by arbitrary rotational force, and neglected orbital tissues except for the globe and ON (Shin et al. 2017). Other FEMs have assumed a fixed ocular rotational center (Grasa and Calvo 2021; Karami et al. 2017), neglected the ON (Al-Sukhun et al. 2006; Karami and Eghtesad 2018; Karami et al. 2017), omitted the orbital connective tissues (Al-Sukhun et al. 2006; Grasa and Calvo 2021; Karami and Eghtesad 2018; Karami et al. 2017), or did not incorporate realistic geometry and EOM contractility (Karami and Eghtesad 2018; Quaia and Optican 2003).

### Optic nerve tethering

The daunting complexity of EOMs and their relationships to orbital connective tissues have motivated the numerous simplifications employed in prior FEMs. However, one physiological situation naturally offers sufficient anatomical simplification to allow a FEM to be compared with uniform functional anatomy in a reproducible situation experimentally observable by magnetic resonance imaging (MRI). Beyond 26° adduction (horizontal rotation toward the midline) from central gaze, the ON, which has idiosyncratically variable sinuosity among individuals, exhausts length redundancy as it becomes uniformly straightened to act as a tether (Clark et al. 2020; Demer 2016; Demer et al. 2017; Suh et al. 2018, 2017). In this straightened configuration, the ON applies tension to the globe, deforming the optic disk and peripapillary tissues (Clark and Demer 2012b; Demer et al. 2017; Demer et al. 2020; Le et al. 2020; Suh et al. 2017), stretches the ON (Clark et al. 2020), and in cases of glaucoma, retracts the eye into its socket (Demer et al. 2017; Demer et al. 2020). We previously described a FEM to evaluate traction exerted by the straight ON by incremental adduction from the 26° threshold of ON tethering to 32° (Shin et al. 2017). This model also assumed ocular rotation about a fixed center and neglected orbital tissues except the ON.

In healthy people, ON tethering in adduction stretches it and translates the eyeball nasally but does not posteriorly (Clark et al. 2020). However, in 35 patients with primary open-angle glaucoma, ON traction was observed in adduction to cause globe retraction that was proposed to damage the ON when repeated numerous times over the lifespan (Clark et al. 2020; Demer 2016; Demer et al. 2017, 2020). Globe translation also alters EOM lever arms sufficiently to alter the effects of EOM surgery designed to correct strabismus (Demer and Clark 2019), and perhaps resolve the seeming paradox that neural commands to EOMs do not otherwise explain discrepancies between vergence and version binocular rotations (Demer and Clark 2019).

### Purpose

The current investigation therefore aimed to implement a more complete FEM of the globe and EOMs that simulates their bilaminar, physiological operation in the context of anatomically and mechanically realistic orbital connective tissues, and without assuming that the globe rotates about an arbitrarily fixed center. Such a model would be testable against actual globe rotation and translation observed by MRI and might then permit realistic inferences about the local mechanical effects on the ON and posterior eye caused by ON tethering in adduction.

## Methods

### Anatomy

**Fig. 1:**
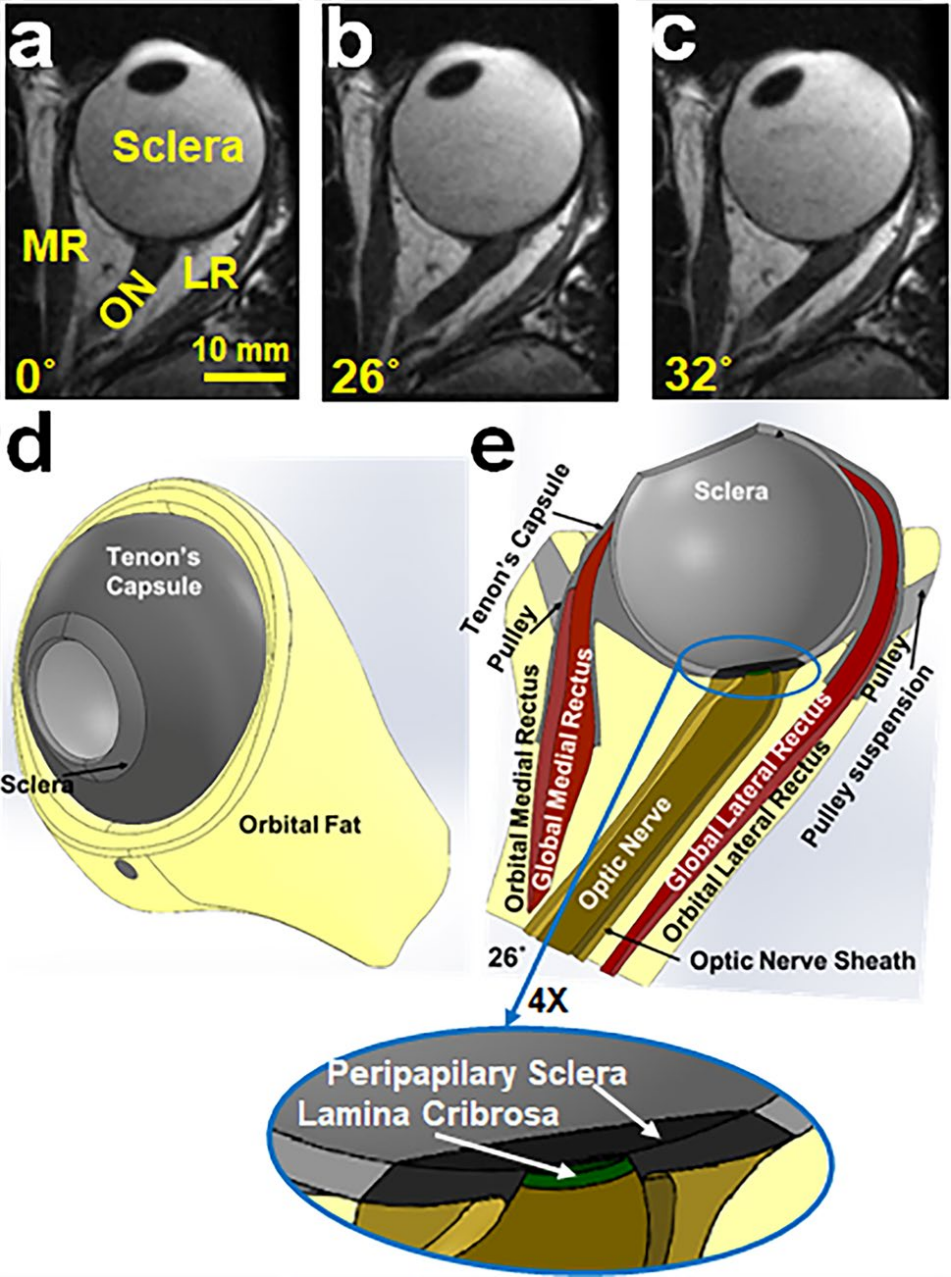
Axial magnetic resonance imaging (MRI) of a left orbit. In **a** central gaze 0°, where ON is sinuous. **b** 26° adduction where ON is first straight; and **c** 32° adduction where elongated ON remains straight. **d** 3D orbit surface rendered in SolidWorks, and horizontally hemisected in **e** showing configuration in 26° adduction

Realistic anatomy of the normal human left orbit was designed to be qualitatively consistent with surface coil magnetic resonance imaging (MRI) using a 1.5T General Electric Signa scanner in five healthy normal adult representatives, similar to our prior studies (Clark et al. 2020; Demer et al. 2017, 2020). These volunteers gave written informed consent prior to participation under a protocol approved by the local Institutional Review Board for Protection of Human Subjects, and in conformity with the Declaration of Helsinki. Imaging was performed with T2 weighting in 2-mm-thick, contiguous axial planes with 100 × 100 mm field of view and 390 μm resolution, and in quasi-coronal planes perpendicular to the long axis of the orbit at with 80 × 80 mm field of with and 312 μm resolution (Demer and Dushyanth 2011). Scans were repeated for three horizontal gaze positions shown in an axial view: central (0°, Fig. 1a), small adduction (~ 26°, Fig. 1b), and large adduction (~ 32°, Fig. 1c).

In this FEM, adduction was simulated by contraction of the dual-layer medial rectus (MR) while the lateral rectus (LR) EOM relaxed. The current model extends an earlier model (Jafari et al. 2021) that simplified each horizontal rectus EOM as a single compartment, rather than as an anatomically realistic bilayer (Demer et al. 2000; Oh et al. 2001) whose dual compartments are mechanically independent (Clark and Demer 2016; Lim et al. 2007; Shin et al. 2012, 2014) and have distinct intramuscular innervation distributions (da Silva Costa et al. 2011; Demer and Clark 2014; Demer et al. 2011; Peng et al. 2010). Implementation of a realistic connective tissue suspension system required that OLs of each EOM insert on their corresponding pulleys. In the current model, the MR and LR physiologically implement horizontal eye rotation, since the MR is the only significant adductor and the LR the principal abductor (Collins et al. 1975). The geometry of sclera, ON and ON sheath is as in the previous model (Jafari et al. 2021). Vertical rectus and oblique rectus EOMs were omitted for simplicity as in previous studies. The MR and LR EOMs are the main drivers of eye rotation (Clark and Stark 1974; Collins et al. 1981, 1975) and are the EOMs operated for surgical treatment of horizontal strabismus (Shin et al. 2012).

A major innovation of this model is the anatomically realistic implementation of connective tissue suspension system around the globe. Globe center is a geometrical notion but not an anatomical structure actually fixed to anything, contrary to prior simplifications (Grasa and Calvo 2021; Iskander et al. 2018; Jafari et al. 2021; Karami et al. 2017; Shin et al. 2017). Rather, globe translation emerges from the FEM due to interaction of EOM forces with a complex suspension system that includes Tenon’s fascia, EOM pulleys, and a cushion of orbital fat (Figs. 1d and e) (Demer and Clark 2019; Moon et al. 2021). The orbital OL of each rectus EOM inserts directly into its pulley to translate it, while the GL passes through the pulley and inserts on the globe to rotate it (Figs. 1e). Initial eye position was set to 26° (small adduction, Fig. 1e that is the average anatomically determined threshold for the transition from ON path redundancy to straightening (Demer et al. 2020), and thus the threshold of ON tethering (Demer et al. 2020).

### Geometry

**Fig. 2:**
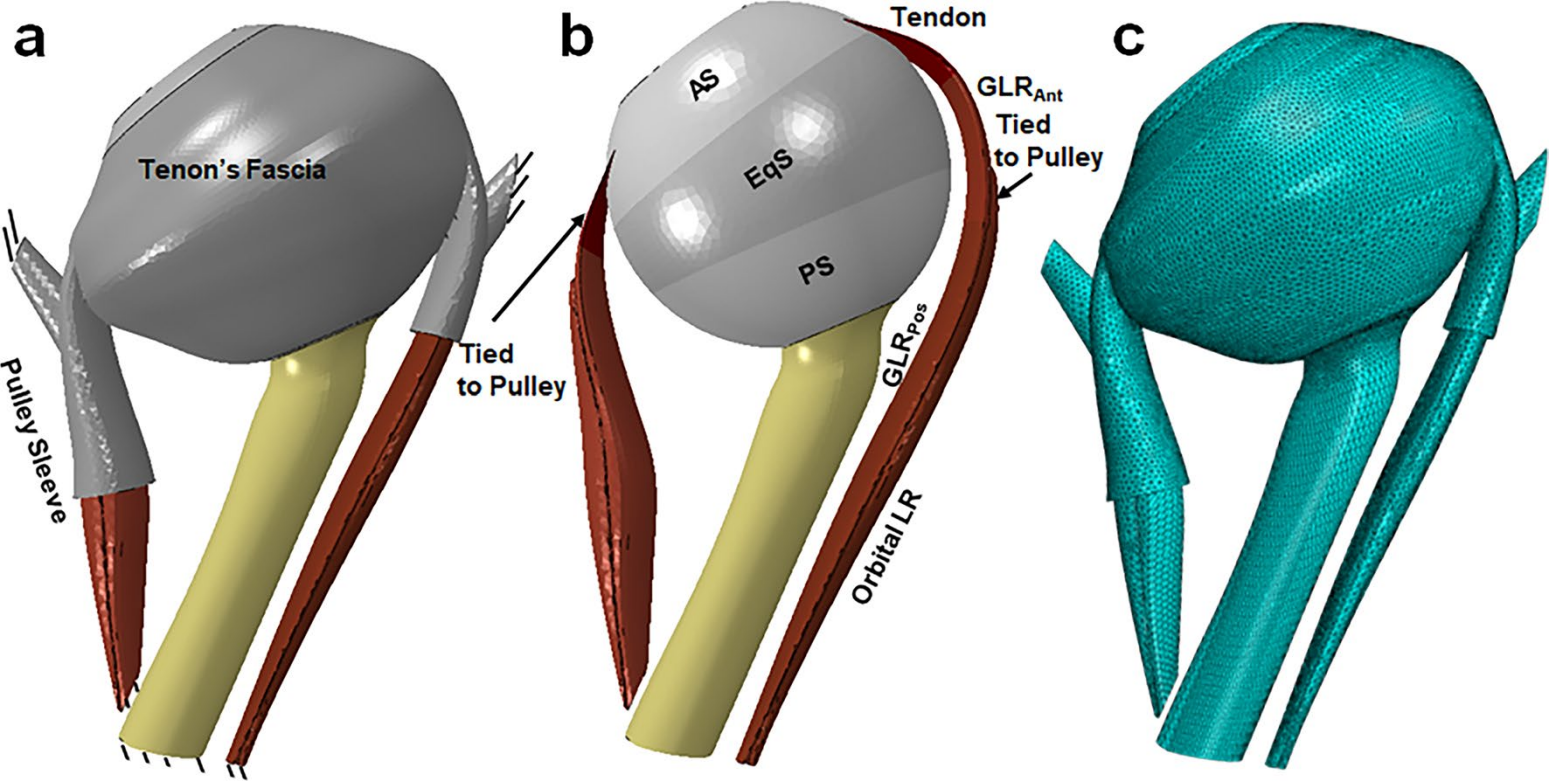
Geometry implemented in ABAQUS. The reference state at 26° adduction, **a** including Tenon’s fascia, pulleys, and connective tissues; and **b** excluding them. The lateral rectus global layer (GLR) is partitioned into three parts: tendon, GLR_Ant_, and GLR_Pos_. The sclera is partitioned into anterior sclera (AS), equatorial sclera (EqS), and posterior sclera (PS). **c** Mesh

The three-dimensional (3D) geometry of the orbit in Fig. 1 is based on high-resolution MRI (Jafari et al. 2021) and published dimensions for various anatomical regions (Li et al. 2019; Norman et al. 2010), including peripapillary sclera (PPS), ON (Elkington et al. 1990), and ON sheath (ONS) (Elkington et al. 1990). The PPS was taken to be an annulus about 0.4 mm thick (Girkin et al. 2017; Vurgese et al. 2012) surrounding the optic disk and having an 8 mm outer diameter. A simple published model of the lamina cribrosa (LC) was included, although the current FEA is not intended to examine LC behavior in detail (Girkin et al. 2017). In this study, 16 quasi-coronal, 2-mm-thick MRI planes perpendicular to the long axis of the orbit were used to define the 3D geometries of the MR and LR EOMs. The ratio of the cross section of OL to GL layers of each EOM, and the orbital fat, was based on coronal plane histologic images of human orbits (Demer et al. 2000; Kono et al. 2002). The geometry of Tenon’s fascia, connective tissues, and pulleys was defined based on the MRI (Demer 2003, 2007). The 3D coordinates of EOM area centroids, as well as their cross-sectional areas, are based on published human data (Clark et al. 2000; Kono et al. 2002). Published locations of GL insertions on the sclera (Apt and Call 1982) and OL insertions on the pulleys (Demer et al. 2000) were implemented. The pulley sleeve is the tubular posterior part of the pulley (Fig. 2a) that readily deforms during EOM contraction and relaxation (Clark and Demer 2009), although the more anterior pulley rings that inflect EOM paths are very stiff. Therefore, the pulley rings were implemented correspondingly stiffer than the sleeves. The LR global layer (GLR) is partitioned into three parts: tendon, anterior muscle belly GLR_Ant_, and posterior muscle belly GLR_Pos_. In this study, in contrast to the previous FEA where the globe rotational center was fixed, there were no constraints on lateral and axial globe translation.

The simulation aimed to implement incremental adduction from the 26° initial state where average ON redundancy is first exhausted, to 32° (Fig. 1c) to characterize the effect of ON tethering. Prestress, which in general cannot be measured in biological systems (Vavourakis et al. 2016), was assumed to be zero at the 26° initial state. A hemisymmetric, 3D model of the reference state was designed using the software package SOLIDWORKS 2020 (Dassault Systèmes SIMULIA Corp., Johnston, RI) and is shown in Figs. 1d and e.

### Material properties

Although most of the extraocular tissue properties are time dependent and anisotropic, to reduce simulation time cost arising from implementing the complex geometry and active EOM contraction, we assumed all model components except EOMs to be homogeneous, isotropic, and hyperelastic, similar to previous studies on biological tissues (Jafari et al. 2021; Miller 2005; Sacks and Sun 2003; Wang et al. 2016; Wex et al. 2015). While peripapillary sclera has known anisotropy distinguishing the circumferential from radial directions (Coudrillier et al. 2013; Karimi et al. 2017) that would probably alter some behavior within the optic disk and lamina cribrosa, these features were not of specific interest in the current FEM, so isotropy was assumed for computational efficiency. In this study, all tissues are assumed to be incompressible, consistent with published data on many biological tissues (Miller 2001; Pitre et al. 2020; Sacks and Sun 2003; Wang et al. 2016; Wex et al. 2015). Published stress–strain curves uniaxial tensile tests from 22 pairs of fresh human ocular specimens (Jafari et al. 2021; Park et al. 2021) were extended by addition of a 2% low toe strain region, and imported into the software package ABAQUS 2020 (Dassault Systèmes SIMULIA Corp., Johnston, RI). The coefficients of reduced polynomial constitutive equations (Table 1) were obtained that resulted in coefficients of determination (R^2^) exceeding 0.99 (Jafari et al. 2021; Park et al. 2021), and have the lowest order compatible with ABAQUS stability. The strain energy function *U* for tissues except rectus EOMs is given by:

1 $$ U = \sum \limits_{n = 1}^{N} C_{i0} \left( {{\bar{\text{I}}}_{1}^{\text{C}} - 3} \right)^{n} + \sum \limits_{n = 1}^{N} \frac{1}{D_{i}}\left( {J - 1} \right)^{2n} $$

where $\bar{\text{I}}_{1}^{\text{C}}$ represents the first invariant of the right Cauchy–Green deformation tensor with the volume change eliminated, and $J$ is elastic volume ratio. For each tissue, an up to 5th-order constitutive model was defined based on its stress–stress curve fitting, as shown in Table 1. ABAQUS assigns ${D}_{i}$ to zero for incompressible tissues, but ABAQUS/Explicit assigns negligible but nonzero compressibility to incompressible material to avert computational instability.

**Table 1:** Hyperelastic reduced polynomial model parameters for passive tissues (Jafari et al. 2021)

Tissue	Model order (N)	Coefficients					
		${C}_{10}\times {10}^{2}$ (MPa)	${C}_{20}$(Mpa)	${C}_{30}\times {10}^{-2}$(Mpa)	${C}_{40}\times {10}^{-3}$(Mpa)	${C}_{50}\times {10}^{-4}$(Mpa)	${D}_{1}$(Mpa)
Lamina cribrosa	4th	12.0	30.0	− 4.2	2.3	–	0.2
Optic nerve	5th	1.1	5.3	2.3	− 3.8	1.7	–
Optic nerve sheath	3rd	2.9	21.5	1.2	–	–	–
Peripapillary sclera	5th	0.4	8.4	2.2	− 3.5	1.5	–
Anterior sclera	3rd	0.5	71.7	2.7	–	–	–
Equatorial sclera	3rd	2.4	17.8	13.7	–	–	–
Posterior sclera	2nd	5.2	28.4	–	–	–	–
Tenon’s fascia	1st	0.085	–	–	–	–	0.48
Pulley sleeve	1st	0.0085	–	–	–	–	4.8
Connective tissue	1st	0.017	–	–	–	–	3.0
Orbital fat	1st	0.00051	–	–	–	–	100
Tendon	Linearly elastic	Young’s modulus, *E* = 50 Mpa					Poisson’s ratio, *ν* = 0.48

Properties of orbital fat are based on data of Schoemaker et al. (Schoemaker et al. 2006). Mechanical properties of the LC were set as justified previously (Jafari et al. 2021). Material properties of Tenon’s fascia, pulleys, and connective tissue are based on the bovine data of Yoo et al. (Yoo et al. 2011) but subject to continuity with adjacent tissues, as there is no available human data for those tissues. Material properties of these tissues are assumed based on the second-order neo-Hookean model in Eq. (1). Tendons were assumed to be linearly elastic with a high Young’s modulus *E*, and Poisson’s ratio *ν* shown in Table 1 as published (Jafari et al. 2021). Intraocular pressure (IOP) and intracranial pressure (ICP) within the ON sheath (ONS) were set to typical normal values of 15 and 10 mmHg, respectively (Berdahl et al. 2008).

### Model implementation

EOM was implemented as a fiber-reinforced material having nonlinear, hyperelastic, and active mechanical behaviors (Jafari et al. 2021), elaborating the 3D skeletal muscle model of Lu et al. (Lu 2010) based on the Hill 3-element model (Hill 1938). The contractile element (CE) represents interaction between actin and myosin, the series elastic element (SEE) represents the elasticity of cross bridging, and the parallel element (PE) represents connective tissue surrounding the EOM (Jafari et al. 2021).

Variables are defined in Table 2 in our previous paper (Jafari et al. 2021) and summarized here for convenience. We employed the published total strain energy for EOM (Jafari et al. 2021):

2 $$U ({\bar{\text{I}}}^{\text{C}}_{1} ,\overline{\lambda }_{f} ,\lambda_{s} ,J) = U_{I} (\bar{\text{I}}_{1}^{\text{C}} ) + U_{f} (\overline{\lambda }_{f} ,\lambda_{s} ) + U_{j} (J),$$

where $U_{I} \left( {\bar{\text{I}}}^{\text{C}}_{1} \right)$ is the strain energy of the isotropic matrix, ${U}_{f}\left({\overline{\lambda }}_{f},{\lambda }_{s}\right)$ is strain energy of fibers within EOMs, and ${U}_{J}\left(J\right)$ is the strain energy related to change of volume. ${\overline{\lambda }}_{f}$ and ${\lambda }_{s}$ are stretch ratios of incompressible fiber and SEE, respectively.

**Table 2:** Variables and functions for extraocular muscle (Jafari et al. 2021)

Variable	Definition
${\lambda }_{s}$	Stretch ratio in series elastic element
$\Delta {\lambda }_{s}$	Stretch increment in series elastic element
${\bar{\text{I}}}^{\text{C}}_{1}$	The first invariant of the right Cauchy–Green strain tensor
$J$	Elastic volume ratio
$\lambda$	Muscle stretch
${\overline{\lambda }}_{f}$	Fiber stretch ratio with the volume change eliminated
${\dot{\lambda }}_{m}$	Stretch rate in contractile element (${\mathrm{s}}^{-1}$)
${\sigma }_{\mathrm{PE}}$	Stress in passive element (Mpa)
${f}_{\mathrm{PE}}$	Normalized function used for ${\sigma }_{\mathrm{PE}}$
${\sigma }_{\mathrm{SEE}}$	Stress in series elastic element (Mpa)
${\sigma }_{\mathrm{CE}}$	Stress in contractile element (Mpa)
${\sigma }_{f}$	Total stress created in muscle (Mpa)
$U({\bar{\text{I}}}^{\text{C}}_{1},{\overline{\lambda }}_{f},{\lambda }_{s},J)$	Strain energy density function in rectus muscle (Mpa)
${U}_{I}({\bar{\text{I}}}^{\text{C}}_{1})$	Strain energy function stored in the isotropic matrix (Mpa)
${U}_{f}({\overline{\lambda }}_{f},{\lambda }_{s})$	Strain energy function stored in the muscle fibers (Mpa)
${U}_{J}(J)$	Strain energy function associated with the volume change (Mpa)
${f}_{t}(t)$	Muscle activation function (Mpa)
${f}_{\lambda }({\overline{\lambda }}_{f})$	Muscle force-stretch function (Mpa)
${f}_{v}\left({\dot{\lambda }}_{m}\right)$	Muscle force–velocity function (Mpa)

Consistent with our published notation, the first term of the strain energy function is (Jafari et al. 2021):

3 $$U_{I} \left( {\bar{\text{I}}}^{\text{C}}_{1} \right) = c\left\{ {{\text{exp}}\left[ {b\left( {{\bar{\text{I}}}^{\text{C}}_{1} - 3} \right)} \right]} \right\} - 1,$$

where ${\text{b}}$ and ${\text{c}}$ are material constants for the isotropic matrix (Table 3). Volumetric strain energy related to compressibility is given by (Jafari et al. 2021):

4 $$U_{J} \left( J \right) = \frac{1}{D}\left( {J - 1} \right)^{2} ,$$

where $D$ is the compressibility constant (Table 1).

**Table 3:** Muscle parameters (Jafari et al. 2021)

Parameter	Definition	Orbital/Global Medial Rectus	Orbital/Global Lateral Rectus	
$b$	Material parameter for isotropic matrix	15.2	15.2	
$c$	Material parameter for isotropic matrix (MPa)	0.1 $\times$ 10^–3^	0.1 $\times$ 10^–3^	
${\sigma }_{0}$	Maximum isometric stress (MPa)	0.04	0.04	
$D$	Compressibility constant (MPa^−1^)	10	10	
$k$	Length ratio SEE: CE	0.3	0.3	
$\alpha$	Material constant in SEE	10	10	
$\beta$	Material constant in SEE (MPa)	0.4 $\times$ 10^–3^	0.4 $\times$ 10^–3^	
${{\lambda }}_{m}^{\mathrm{min}}$	Minimum stretch rate (s−1)	− 10	− 10	
${k}_{c}$	Shape parameter in force–velocity function	5	5	
${k}_{e}$	Shape parameter in force–velocity function	5	5	
$d$	Offset of the eccentric function	1.5	1.5	
${t}_{0}$	Activation time (s)	0	0	
${t}_{1}$	Deactivation time (s)	0.4	0	
$S$	Exponential factor in activation function	100	100	
$A$	Material parameter for stress in PE	4	4	
${n}_{1}$	Activation level before and after activation	0.6	0	
${n}_{2}$	Activation level during activation	1	0	
${\lambda }_{\mathrm{opt}}$	Optimal fiber stretch	1.05	1.05	
${f}_{\mathrm{PE}}^{0}$	Initial normalized force within PE	0	0.2	
${m}_{1}$	First component of unit vector along the muscle fiber direction	0	0	
			Orbital/ Global Posterior	Anterior
${m}_{2}$	Second component of unit vector along the muscle fiber direction	0.25	0.175	− 0.43
${m}_{3}$	Third component of unit vector along the muscle fiber direction	− 0.97	0.984	0.902
${\lambda }_{f}^{0}$	Initial stretch for muscle	0.02	1.03	1.01

The second term of Eq. (2) is:

5 $$U_{f} \left( {\overline{\lambda }_{f} ,\lambda_{s} } \right) = \mathop \smallint \limits_{1}^{{\overline{\lambda }_{f} }} \left[ {\sigma_{{{\text{SEE}}}} \left( {\lambda ,\lambda_{s} } \right) + \sigma_{{{\text{PE}}}} \left( \lambda \right)} \right]{\text{d}}\lambda .$$

$\sigma_{{{\text{SEE}}}} \left( {\lambda ,\lambda_{s} } \right)$ and $\sigma_{{{\text{PE}}}} \left( \lambda \right)$ are the stresses in the SEE and PE, respectively. $\sigma_{{{\text{SEE}}}}$ is expressed as:

6 $${\phantom {i}}^{t + \Delta t} \sigma_{\text{SEE}} = e^{{\alpha \Delta \lambda_{s} }} \left( {\beta \left[ {e^{{\alpha ({\phantom {i}}^{t} \lambda_{S} - 1)}} - 1} \right] + \beta } \right) - \beta ,$$

where $\alpha$ and $\beta$ are the material constants of the SEE.

Stress in the CE is represented by:

7 $$^{t + \Delta t} \sigma_{{{\text{CE}}}} = \sigma_{0} \cdot f_{t} \left( {t + \Delta t} \right) \cdot f_{\lambda } \left( {\overline{\lambda }_{f} } \right) \cdot f_{v} \left( {\dot{\lambda }_{m} } \right),$$

where $\sigma_{0}$ is the maximum isometric stress, and ${f}_{t}(t)$ is EOM activation function (Jafari et al. 2021; Kojic et al. 1998):

8 $${f}_{t}\left(t\right)=\left\{\begin{array}{c}{n}_{1}, if t<{t}_{0}\\ {n}_{1}+\left({n}_{2}-{n}_{1}\right)\cdot {h}_{t}\left(t,{t}_{0}\right), if {t}_{0}<t<{t}_{1}\\ {n}_{1}+\left({n}_{2}-{n}_{1}\right)\cdot {h}_{t}\left({t}_{1},{t}_{0}\right)-\left[\left({n}_{2}-{n}_{1}\right)\cdot {h}_{t}\left({t}_{1},{t}_{0}\right)\right]\cdot {h}_{t}\left(t,{t}_{1}\right), if t>{t}_{1}\end{array}\right.,$$

where ${n}_{1}$ is the level of EOM activation before and after activation, and ${n}_{2}$ is the level during activation. ${t}_{0}$ is the time when activation occurs, and ${t}_{1}$ is the time when the deactivation occurs. ${h}_{t}\left({t}_{i},{t}_{j}\right)$ is given by:

9 $$h_{t} \left( {t_{i} ,t_{j} } \right) = \left\{ {1 - {\text{exp}}\left[ { - S\cdot\left( {t_{i} - t_{j} } \right)} \right]} \right\}.$$

where $S$ is an exponential parameter. ${t}_{i}$ varies between $t$ or ${t}_{1}$, and ${t}_{j}$ varies between ${t}_{0}$ or ${t}_{1}$ in Eq. (8).

In Eq. (7),${f}_{\lambda }({\overline{\lambda }}_{f})$ is the force-stretch function of EOM:

10 $${f}_{\lambda }\left({\overline{\lambda }}_{f}\right)=  \left\{\begin{array}{ll}0, if \frac{{}{}^{t}{\overline{\lambda }}_{f}}{{\lambda }_{\mathrm{opt}}}<0.4\\ 9{(\frac{{}{}^{t}{\overline{\lambda }}_{f}}{{\lambda }_{\mathrm{opt}}}-0.4)}^{2}, if 0.4\le \frac{{}{}^{t}{\overline{\lambda }}_{f}}{{\lambda }_{\mathrm{opt}}}<0.6\\ 1-4{\left(1-\frac{{}{}^{t}{\overline{\lambda }}_{f}}{{\lambda }_{\mathrm{opt}}}\right)}^{2}, if 0.6\le \frac{{}{}^{t}{\overline{\lambda }}_{f}}{{\lambda }_{\mathrm{opt}}}<1.4 \\ 9{\left(\frac{{}{}^{t}{\overline{\lambda }}_{f}}{{\lambda }_{\mathrm{opt}}}-1.6\right)}^{2}, if 1.4\le \frac{{}{}^{t}{\overline{\lambda }}_{f}}{{\lambda }_{\mathrm{opt}}}<1.6\\  0, if \frac{{}{}^{t}{\overline{\lambda }}_{f}}{{\lambda }_{\mathrm{opt}}}\ge 1.6\end{array} \right\}.$$

In Eq. (10), ${\lambda }_{\mathrm{opt}}$ is a constant defined as optimal fiber stretch (Table 3).

${f}_{v}\left({\dot{\lambda }}_{m}\right)$ in Eq. (7) is the force–velocity function of EOM:

11 $${f}_{v}\left({\dot{\lambda }}_{m}\right)=\left\{\begin{array}{ll}{c}\frac{1-\frac{{\dot{\lambda }}_{m}}{{\dot{\lambda }}_{m}^{\mathrm{min}}}}{1+\frac{{k}_{c}{\dot{\lambda }}_{m}}{{\dot{\lambda }}_{m}^{\mathrm{min}}}}, if {\dot{\lambda }}_{m}\le 0\\ d-\left(d-1\right)\frac{1+\frac{{\dot{\lambda }}_{m}}{{\dot{\lambda }}_{m}^{\mathrm{min}}}}{1-\frac{{k}_{c}{k}_{e}{\dot{\lambda }}_{m}}{{\dot{\lambda }}_{m}^{\mathrm{min}}}}, if {\dot{\lambda }}_{m}>0\end{array}\right\},$$

where ${\dot{\lambda }}_{m}$ is the stretch rate in the CE, ${\dot{\lambda }}_{m}^{\mathrm{min}}$ is the minimum stretch rate, ${k}_{c}$ and ${k}_{e}$ are the shape constants, and $d$ is the offset of the eccentric function (Jafari et al. 2021). Based on the relationship between the stresses in the SEE and PE ${\phantom {i}}^{t+\Delta t}{\sigma }_{\mathrm{SEE}}={\phantom {i}}^{t+\Delta t}{\sigma }_{\mathrm{CE}}$ and referring to Eq. (6), the unknown variable $\Delta {\lambda }_{s}$ can be defined (Lu et al. 2010). Stress in the PE is

12 $${\phantom {i}}^{t + \Delta t} \sigma_{\text{PE}} \left( {\overline{\lambda }_{f} } \right) = \sigma_{0} f_{\text{PE}} \left( {{\phantom {i}}^{t + \Delta t} \overline{\lambda }_{f} } \right),$$

with

13 $$f_{{{\text{PE}}}} \left( {\overline{\lambda }_{f} } \right) = \left\{ {\begin{array}{ll}{ A\left( {\overline{\lambda }_{f} - 1} \right)^{2} , {\text{ if}} \overline{\lambda }_{f} > 1} \\ { 0, {\text{otherwise}} } \\ \end{array} } \right.,$$

where $A$ is a material constant.

Material constants $b$,$c$, $\alpha ,$ and $\beta$ were obtained as previously described (Gao et al. 2014; Humphrey and Yin 1987; Jafari et al. 2021) using the “cftool” function in MATLAB R2019a (MathWorks, Natick, MA).

### FEM simulation

The model (Fig. 1d) was designed in SOLIDWORKS and simulated in ABAQUS/Explicit (Fig. 2a). Contraction of EOM was performed by a VUMAT (Jafari et al. 2021). The LR is modeled in anterior (GLR_Ant_) and posterior regions (GLR_Pos_, Fig. 2a and b) due to differing fiber orientation as defined in a VUMAT. As previously, initial EOM stretch (${\lambda }_{f}^{0}$) and fiber directions (m_2_ and m_3_) were separately defined according to the stretch fraction and fiber direction within GLR_Ant_, GLR_Pos_, and the OLR (Jafari et al. 2021). A pre-stretch ${\lambda }_{f}^{0}$ and a normalized initial PE force *f*_PE_^0^ were applied to the LR, whereas a pre-stretch ${\lambda }_{f}^{0}$ was applied to the MR at 26° (Table 3). Active LR force was neglected as it is minimal for adduction exceeding 26° (Clark and Stark 1974; Robinson et al. 1969). The MR developed active force at the 26° adduction initial position. Since prestress cannot be known in passive tissues (Vavourakis et al. 2016), other extraocular tissues were assumed to be stress free at 26°. A mass scaling factor of 100 was used to reduce simulation time. The ratio of kinetic energy to internal energy for the whole model was tracked to remain less than 5%.

The anterior terminations of the LR and MR OLs are tied to the inner layers of their respective pulleys, while GLs of the LR and MR pass through their pulleys and insert on the sclera (Fig. 2b). Origins of the LR and MR EOMs are fixed at the orbital apex (Fig. 2a) where the extraocular muscles originate (Sevel 1986).

All elements in ABAQUS are defined as explicit tetrahedra. Average optimized mesh was set to about 0.8 mm for EOMs and 0.6 mm near the optic disk (Fig. 2c), sufficiently fine to obtain consistent results. The model contains approximately one million elements. Mesh convergence testing was done by using finer mesh sizes around half current sizes for EOMs, resulting in only about 0.3% change in maximum principal strain. Further reduction of mesh sizes would increase computation time prohibitively beyond the several days required for the chosen mesh. The complete model with orbital fat rendered transparent is shown in Fig. 2c.

We simulated three situations: Case 1, incorporating all components without constraint on globe translation (Fig. 1d); Case 2, identical to Case 1 but with globe rotation constrained to its fixed center prohibiting translation; and Case 3, similar to Case 1 but omitting orbital fat. Computing time for each simulation was 3–4 days using a six-processor Intel(R) Core (TM) i7-4930K CPU running at 3.40GHz with 64 Gb random access memory.

## Results

### Strain distribution in rectus muscles (Case 1)

**Fig. 3:**
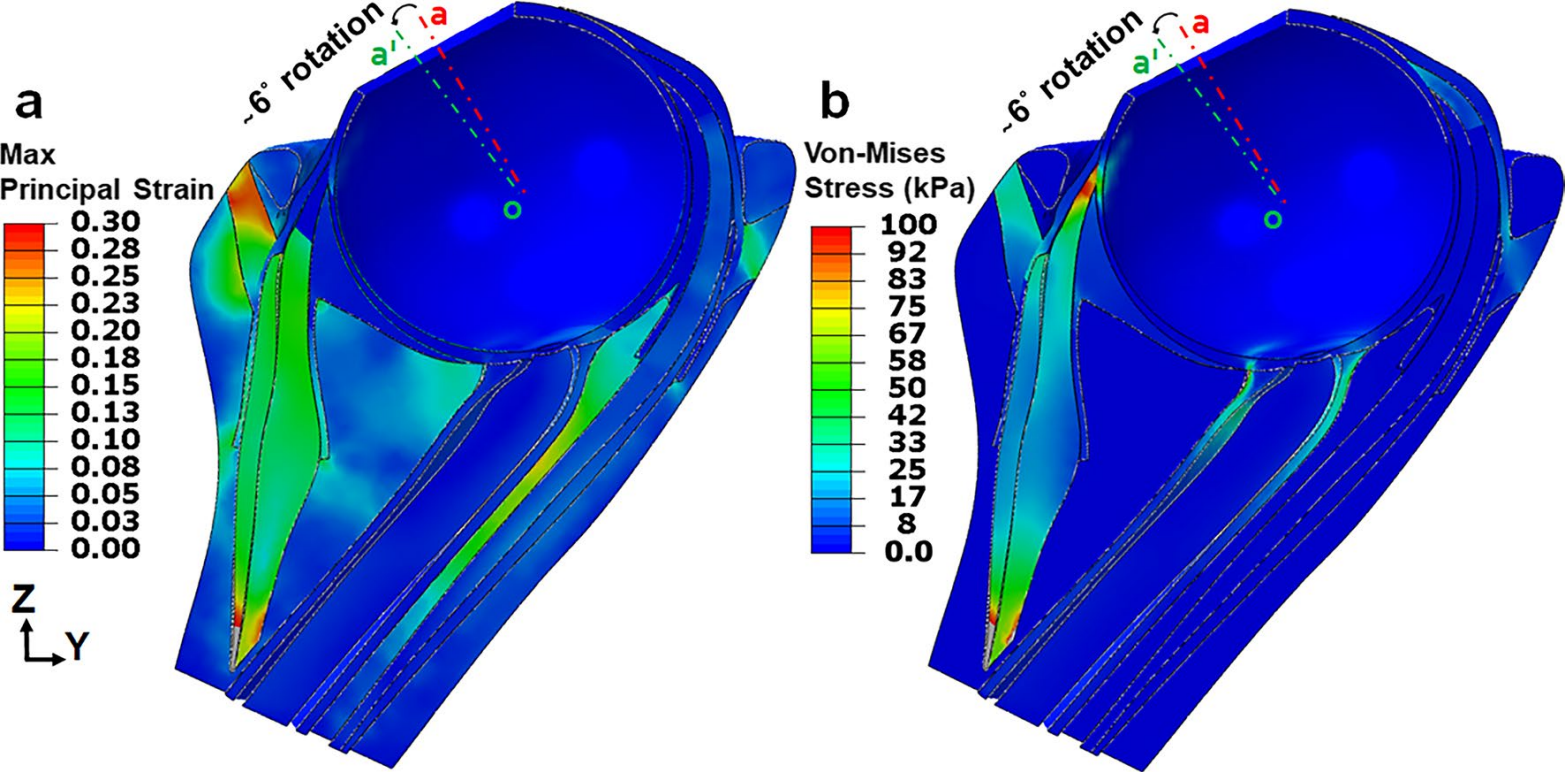
Principal logarithmic strain and von-Mises stress distribution within rectus muscles, connective tissues, optic nerve, and optic nerve sheath from 26 to 32° adduction for Case 1. **a** Maximum principal strain, ${\varepsilon }_{max}$. **b** Von-Mises stress, ${\sigma }_{v}$ color map. Red and green dashed lines (oa and oa′) indicate eye orientation in the reference and adducted states, respectively. Gray color in MR origin is out of range but not in an area of interest

**Fig. 4:**
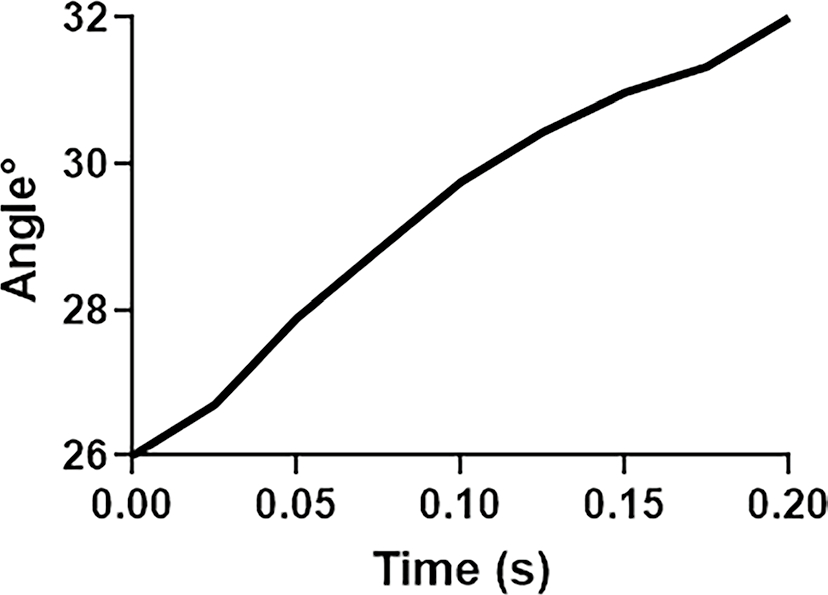
Time course of simulated adduction from reference state 26° to nearly 32° adduction for Case 1

Under active contraction of both MR layers, the simulation produced approximately 6° adduction beyond the initial position of 26° (red dashed line oa) to 32° (green dashed line oaʹ, Fig. 3) in 200 ms, with 30°/s average velocity (Fig. 4). Maximum principal strain (${\varepsilon }_{\mathrm{max}}$) was 0.13 along the transverse direction (thickness) of the MR, and was about 0.06 in the LR. For comparison, in the previous model that constrained globe center, computed ${\varepsilon }_{\mathrm{max}}$ in the LR was much larger at 0.13 (Jafari et al. 2021) (Fig. 3a).

### Von-Mises stress (Case 1)

Maximum von-Mises stress, ${\sigma }_{v}$ was around 30 kPa within EOM bellies (Fig. 3b). These stresses are much lower than EOM stiffness of about 10 MPa. Stress was approximately 90 kPa within the anterior ONS near the ON head. Local stress and strain distributions within orbital fat are not of interest to simulate, as these have never been measured and orbital fat behaves like a semi-fluid suspension.

### Effect of orbital fat on globe translation

**Fig. 5:**
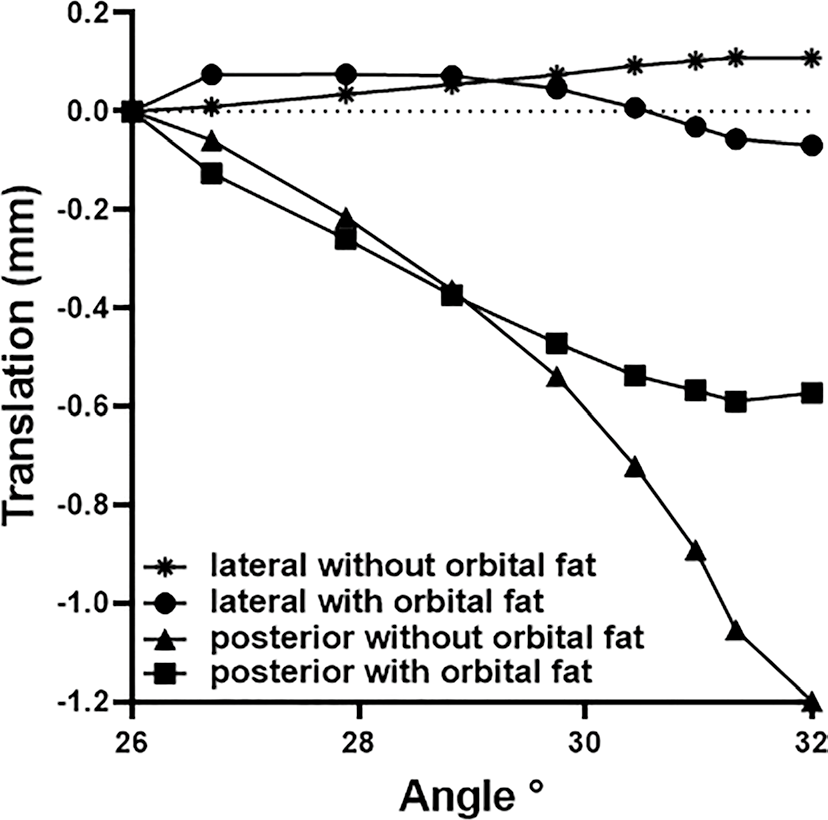
Simulated lateral and posterior globe translation during rotation from reference state 26° to nearly 32° adduction, with (Case 1), and without inclusion (Case 3) of orbital fat

For Case 1 that includes the connective tissue suspensory system and orbital fat, the globe translated 0.1 mm medially and 0.52 mm posteriorly during 6° incremental adduction (Fig. 5). For Case 3, omitting orbital fat but including the other suspensory tissues, the globe translated 0.1 mm laterally but 1.2 mm posteriorly (Fig. 5).

### Muscle and tissue forces

**Fig. 6:**
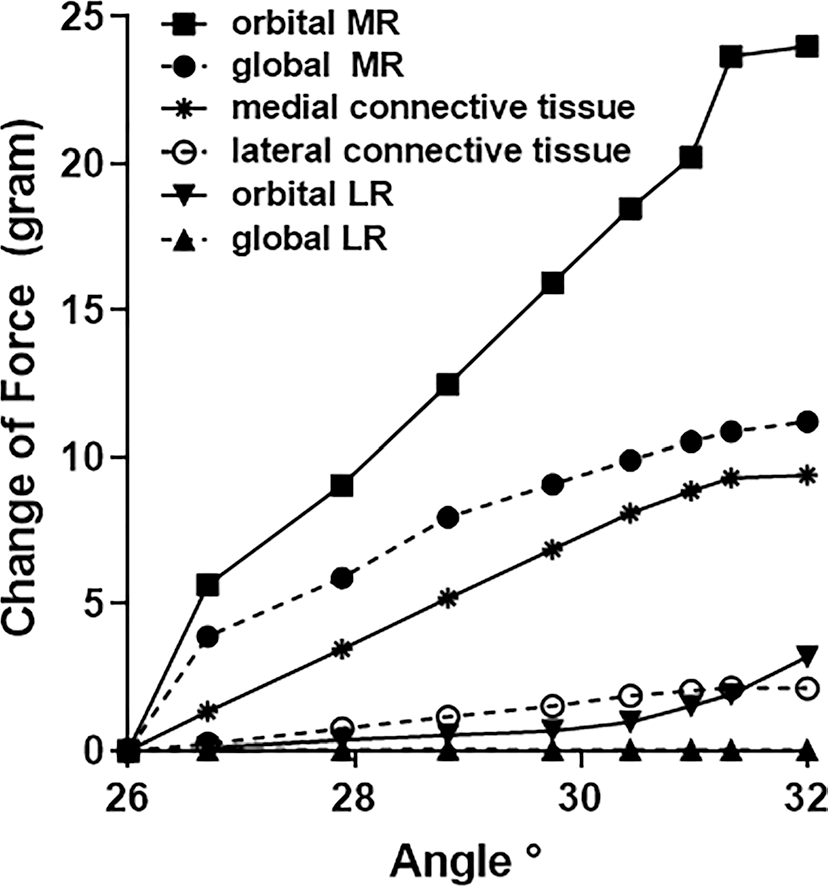
Total gm-f in the global layer (GL) and orbital layer (OL) of the medial rectus (MR) and lateral rectus (LR) muscles, as well as the medial and lateral connective tissues, during 6° adduction from 26 to 32° for Case 1

For comparability with published data, Fig. 6 depicts the total gram force (gm-f) in the GL and ML of the horizontal rectus EOMs, as well as corresponding connective tissues, during the 6° incremental adduction for Case 1. Not surprisingly since it is the contracting actuator of adduction, forces in both MR layers exceed those in both LR layers and the connective tissues. The MR OL applied about 24 gm-f to the pulley upon reaching 32° adduction. Since the LR is relaxed during adduction, LR force was much lower, approximately 3 gm-f in its OL and near zero in its GL. As the MR muscle contracted, it exerted 9 gm-f on the medial connective tissues, while force in lateral connective tissue was about 2 gm-f.

**Fig. 7:**
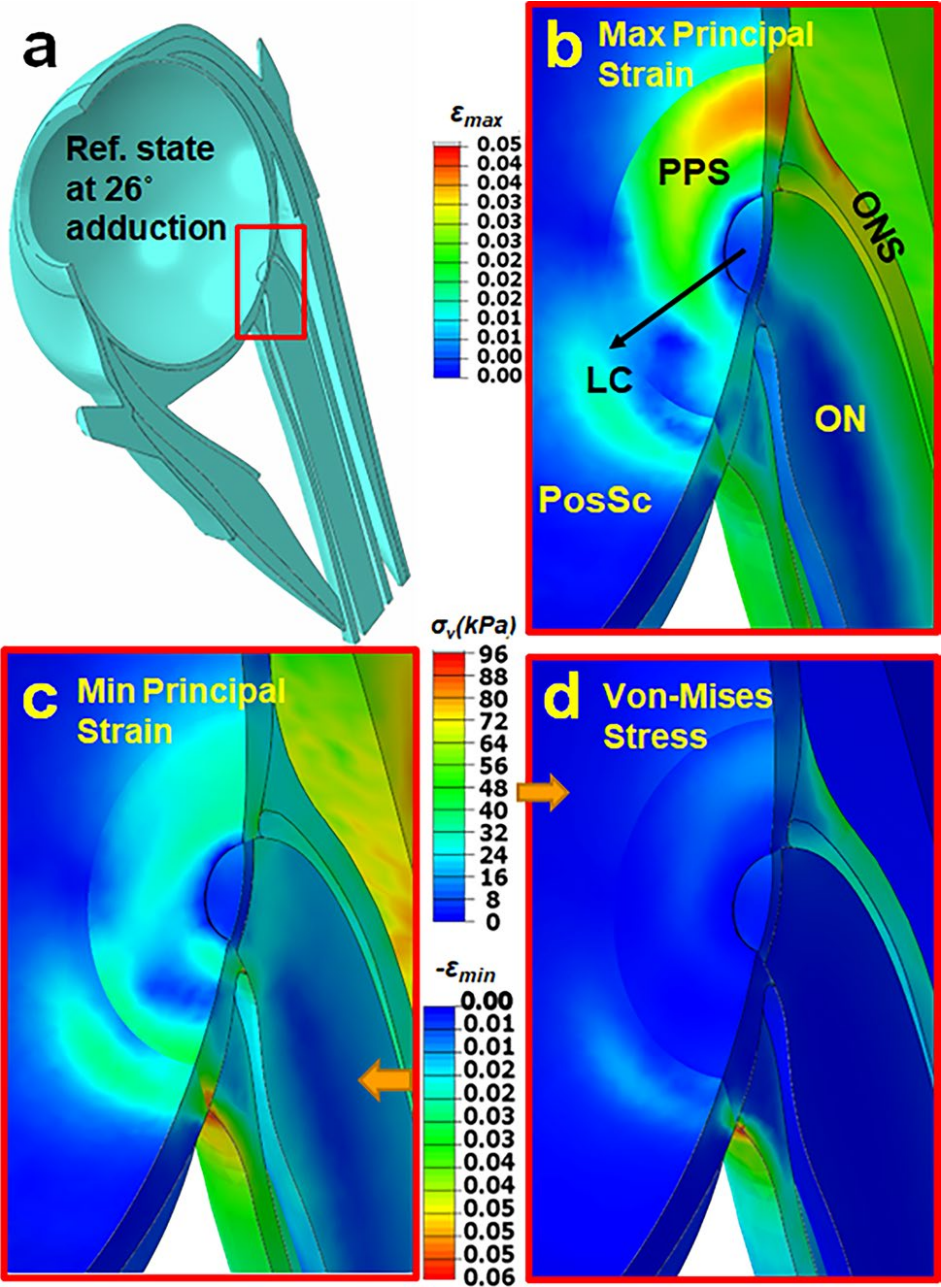
Simulation for the complete model Case 1 of 6° incremental adduction beyond threshold optic nerve tethering at 26°. **a** Reference state 26° adduction. The region within red rectangle in panel **a** is magnified 4 × in panels **b–d**. Color map shows maximum (**b**, ${\varepsilon }_{\mathrm{max}}$) and minimum (**c**, ${\varepsilon }_{\mathrm{min}}$) principal strains, and von-Mises stress (**d**, ${\sigma }_{v}$). Orbital fat is transparent for clarity

Maximum (${\varepsilon }_{\mathrm{max}}$) and minimum (${\varepsilon }_{\mathrm{min}}$) principal strains and von-Mises Stress (${\sigma }_{v}$) distributions in the posterior eye at 32° adduction are illustrated in Fig. 7 for Case 1. As shown in Fig. 7b, ${\varepsilon }_{\mathrm{max}}$ reached 0.05 within the lateral PPS and ONS, and about 0.03 within the ON. Unlike ${\varepsilon }_{\mathrm{max}}$ that was maximal in the lateral PPS and ONS, ${\varepsilon }_{\mathrm{min}}$ reached its maximum of 0.06 at the junction of ONS and PPS (Fig. 7c). Average ${\sigma }_{v}$ was 50 kPa in the temporal side of ONS and 20kPa in PPS, except at the junction between the two, where discontinuity of tissue property differences caused ${\sigma }_{v}$ to be 100kPa as an edge artifact of the junction between these tissues (Fig. 7d).

### Translation constraint

**Fig. 8:**
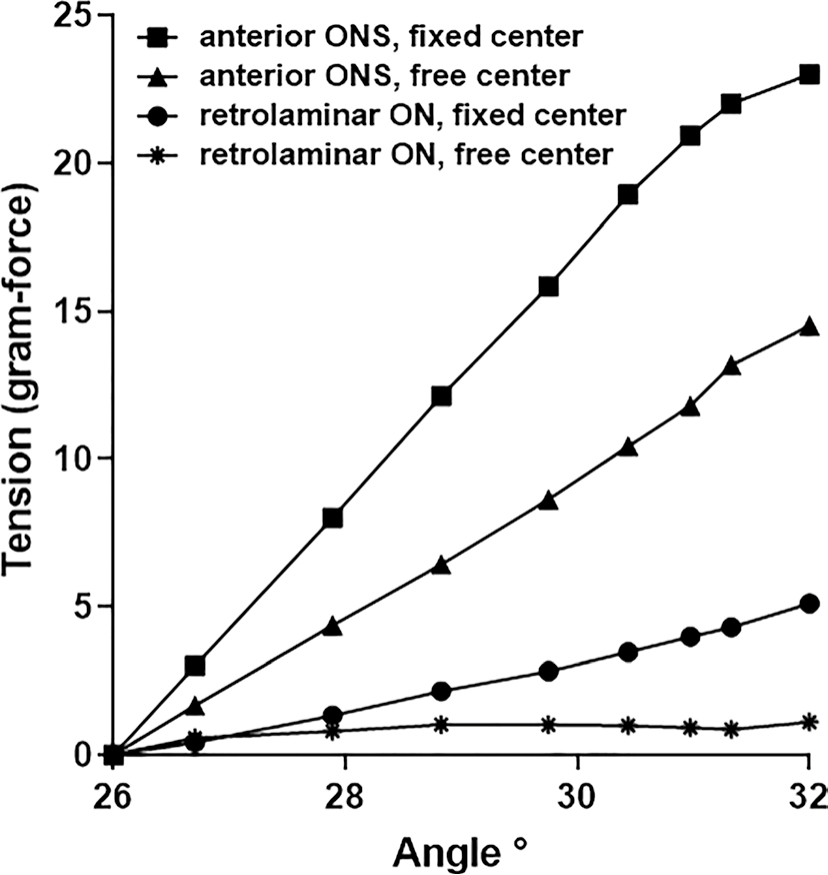
Change of force in the retrolaminar ON and ONS with (Case 1) and without (Case 2) globe translation constraint during rotation from reference state 26° to nearly 32° adduction

Forces within the retrolaminar ON, defined as the ON region immediately posterior to the LC with a thickness similar to it, and anterior ONS are illustrated in Fig. 8, in alternative cases when globe center is free to translate (Case 1), versus fixed (Case 2). When translation was permitted, after 32° adduction retrolaminar ON tension reached 1 gm-f and ONS tension 13 gm-f. However, with globe center constrained, retrolaminar ON tension increased much more to 5 gm-f and ONS tension to 23 gm-f (Fig. 8).

## Discussion

### Realism

This FEM of an anatomically realistic human eye and orbit demonstrates plausible forces during adduction tethering from 26 to 32° as driven by physiologically realistic bilaminar horizontal rectus EOM activation, with realistic passive connective tissues obviating unrealistic prior constraints on ocular translation (Crane et al. 2005; Fuller 1996; Lefevre et al. 1992; Moschner and Zangemeister 1993; van Wetter and van Opstal 2008). Unlike the previous model where rectus EOMs were considered unicompartmental (Jafari et al. 2021), in the current FEM the MR and LR were realistically modeled as bilaminar, with each OL inserting on its pulley connective tissue ring while the GL transits the pulley to insert on the globe (Demer 2003, 2007). Contrary to the suggestion of Schutte et al. (Schutte et al. 2006), the current FEM allowed sliding between the posterior eye and orbital fat. Constraining ocular motility with fat alone  may cause failure of ocular motility because the eye requires additional support from the connective tissue pulley system. Inclusion of orbital fat to the pulley system reduced posterior globe translation when it was tethered by the ON in adduction. Unlike Schutte et al.’s FEM in which EOM contraction was implemented through thermal expansion and contraction (Schutte et al. 2006), the present FEM was driven by physiological contractile behavior of EOMs.

### Translation

The current model highlights the importance of constraints on globe translation during ON tethering in adduction. When globe translation during rotation is artificially constrained to compel rotation about its center, the FEM predicts that adduction to 32° causes about 5 gm-f tension in the ON, and 23 gm-f in the ONS. When the globe is suspended by realistic connective tissues as in the current model, its center is free to translate posteriorly and nasally, so that during the same adduction tension reaches only about 1 gm-f within the anterior ON and 15 gm-f in the ONS. The magnitudes of these tensions are of order similar to calculations by Wang et al. (Wang et al. 2017) for adduction, and experimentally measured in human rectus EOMs (Collins et al. 1981).

### Muscle forces

The current FEM predicts MR and LR forces with magnitudes consistent with previous published experimental data (Collins et al. 1981, 1975; Karami et al. 2017; Miller and Robinson 1984; Robinson 1964, 1975; Robinson et al. 1969). The force simulated by the FEM in the OL of the MR reached ~ 24 g during 6° adduction, while the force in the GL of the MR reached ~ 12 g. Therefore, the total simulated MR force of 36 g is in agreement with experimental observations in human MR where measured force magnitude was ~ 25–46 g at 32° adduction (Collins et al. 1981, 1975). The FEM predicts that forces in both layers of the relaxing LR to be very small, averaging about 2–3 g. Due to the MR contraction, the medial connective tissue experiences about 10 g of tension as its elasticity resists MR shortening.

### Connective tissue and fat

The relative contributions of orbital connective tissue versus fat have been debated (Schoemaker et al. 2006; Schutte et al. 2006; Yoo et al. 2011), with Schutte et al. claiming based on a FEM and extensive tying of orbital fat that the fat alone suffices to support the globe and stabilize EOM paths, without pulleys (Schoemaker et al. 2006; Schutte et al. 2006). While in realistic case, eye globe is sliding over orbital fat; therefore, the suspensory system including Tenon’s fascia and connective tissue pulleys may play an important role in addition to the orbital fat (Demer 2003, 2007; Grasa and Calvo 2021; Iskander et al. 2018). The FEM predicted posterior translation during adduction tethering to double when orbital fat is omitted. With realistic inclusion of orbital fat, the FEM predicts ~ 0.52 mm posterior globe translation during adduction tethering, which is only modestly larger than observed by MRI. In healthy people, 26° adduction causes about 0.2 mm globe retraction, but almost no additional retraction during further adduction to 32° (Demer et al. 2020). The greater globe retraction predicted by the current FEM may be due to omission of the oblique EOMs that insert on the posterior globe, and hence exert forces opposing globe retraction. These oblique EOMs probably contribute to globe support during horizontal duction.

During contraction, the OL and GL of the MR shorten longitudinally as they bulge radially during adduction. This bulging was more obvious in the GL and is consistent with MRI that shows an increase in the cross sections of contracting human EOMs (Clark and Demer 2012b).

### Ocular loading

Because of ON tethering, incremental adduction from 26 to 32° concentrates loading on the posterior globe. Minimum principal strain was greatest at the junction of the posterior eye and ONS. It has been suggested that high strain on the posterior eye in healthy young eyes does not damage them because the tissue is compliant as MRIs also show an overall level of 5% strain in the ON during adduction (Clark et al. 2020; Le et al. 2020; Lim and Demer 2023). When globe translation is not constrained, our FEM predicts average von-Mises stress to reach ~ 50 kPa within temporal side of ONS; this stress concentration might damage the ON when repeated over numerous eye movement cycles (Clark et al. 2020; Demer 2016; Suh et al. 2018, 2017). Average stress in this region was predicted by FEM to be ~ 80 kPa when globe is fixed (Jafari et al. 2021). These stress magnitudes in the posterior eye are comparable to those computed by Wang et al. at ~ 200 kPa after 13° adduction from primary gaze while the globe center is fixed (Wang et al. 2017). Therefore, posterior translation concentrated higher stress within the ONS. Unlike an earlier FEM that constrained globe rotation to its center and predicted maximum absolute strains and stresses to occur in the LC (Jafari et al. 2021), in the current FEM these maximal concentrations were within the PPS and ONS. Therefore, it appears that posterior globe translation reduces strain and stress in and near the LC and transfers them to the PPS and ONS. Future realistic simulation of dynamic eye movements will require realistic viscoelastic parameterization of EOMs and passive orbital tissues for which data are currently limited or unavailable.

### Limitations

While the current FEM is the most detailed and computationally intensive model to date, some simplifications persist. The model is hemisymmetrical and omits the vertical rectus and oblique EOMS. A more complete model including these cyclovertical EOMs could not be hemisymmetrical, and therefore would require a large increase in the number of volume elements that would increase already substantial computational time. While most materials were assumed hyperelastic, it is known that ocular tissues typically have time-dependent viscoelastic behavior (Bisplinghoff et al. 2009; Downs et al. 2003; Palko et al. 2011; Yoo et al. 2011) that were not fully considered by the limited dynamics of the current FEM. In particular, orbital fat included in previous studies (Schoemaker et al. 2006; Yoo et al. 2011) is better to be modeled as viscoelastic material or viscous fluid. Furthermore, the LC and ONS have anisotropic structures that may change local stress and strain distributions (Coudrillier et al. 2013; Downs et al. 2008; Girard et al. 2009; Karimi et al. 2021) within these tissues. Prestress within the tissues cannot be known (Vavourakis et al. 2016), and could influence FEM behavior and creates some errors, particularly for hyperelastic tissues. Regardless of the limitations, the current FEM model is useful in understanding the critical role of the ocular suspension system.

## Data Availability

Not applicable to this theoretical study.

## Code Availability

The model has been designed in SOLIDWORKS and four cases have been simulated in ABAQUS. Postprocessing was done in ABAQUS and MATLAB. Files are available at: https://zenodo.org/record/7306040#.Y2reWHbMKUk
